# Kinetics of Plasma Cell-Free DNA under a Highly Standardized and Controlled Stress Induction

**DOI:** 10.3390/cells12040564

**Published:** 2023-02-09

**Authors:** Benedict Herhaus, Elmo Neuberger, Ema Juškevičiūtė, Perikles Simon, Katja Petrowski

**Affiliations:** 1 Medical Psychology and Medical Sociology, University Medical Center of the Johannes Gutenberg University Mainz, 55128 Mainz, Germany; 2 Department of Sports Medicine, Disease Prevention and Rehabilitation, Johannes Gutenberg University Mainz, 55128 Mainz, Germany; 3 Institute of Sport Science and Innovations, Lithuanian Sports University, 44221 Kaunas, Lithuania

**Keywords:** circulating cell-free DNA (cfDNA), Trier Social Stress Test (TSST), psychological stress

## Abstract

Psychological stress affects the immune system and activates peripheral inflammatory pathways. Circulating cell-free DNA (cfDNA) is associated with systemic inflammation, and recent research indicates that cfDNA is an inflammatory marker that is sensitive to psychological stress in humans. The present study investigated the effects of acute stress on the kinetics of cfDNA in a within-subjects design. Twenty-nine males (mean age: 24.34 ± 4.08 years) underwent both the Trier Social Stress Test (TSST) and a resting condition. Blood samples were collected at two time points before and at 9 time points up to 105 min after both conditions. The cfDNA immediately increased 2-fold after the TSST and returned to baseline levels after 30 min after the test, showing that a brief psychological stressor was sufficient to evoke a robust and rapid increase in cfDNA levels. No associations were detected between perceived stress, whereas subjects with higher basal cfDNA levels showed higher increases. The rapid cfDNA regulation might be attributed to the transient activation of immune cells caused by neuroendocrine-immune activation. Further research is required to evaluate the reliability of cfDNA as a marker of neuroendocrine-immune activation, which could be used for diagnostics purposes or monitoring of treatment progression.

## 1. Introduction

All organisms, including humans, maintain a complex dynamic equilibrium, which is constantly challenged by internal or external stressors [1]. Acute psychological stress, lasting for minutes, challenges the homeostasis and activates a complex interplay of nervous, endocrine, and immune mechanisms [2]. The threshold for the involvement of each of the mechanisms is highly individualized and is related to the genetic, epigenetic, and environmental background of individuals [3]. Long-term stress is a predicting factor for developing physiological symptoms, including depression, anxiety, and schizophrenia [4]. Moreover, psychological stress is an independent risk factor for coronary heart disease associated with increased inflammation [5]. Modern societies seem to be particularly prone to stress-related disorders [1], and stress-related mental disorders have been constantly on the rise in recent years, entailing a tremendous societal socio-economic burden [6].

Over the recent years, there has been a growing interest in finding standardized and validated physiological and/or biological markers linked to stress as predictors for defining an individual’s ability to cope with stress or withstand and adapt to adverse and traumatic events [7]. As reviewed by Walker et al. [7], a number of neurobiological candidate markers had been studied in laboratory or epidemiological studies [7]. The approaches included physiological measures, such as cardiovascular reactivity; biochemical measures, such as cortisol; and immunological measures, such as cytokines or cell reactivity [7].

Acute stress impacts immune homeostasis at multiple levels, and the inflammatory responses were studied with various immune markers, including cytokines, such as IL-6, IL-10, IL-ß, and TNF-a [8]. More recently, circulating cell-free DNA (cfDNA) has been suggested to be a relevant marker displaying acute stress [9], as it is a potential biomarker for psychiatric disorders, including schizophrenia [10], and it shows responses to stress reduction interventions [11]. Consisting of a mixture of DNA from different cells and tissues within the human body [12], cfDNA is constantly released into circulation [13]. Elevated levels of cfDNA have been found in a plethora of inflammation-associated pathophysiological conditions, such as sepsis [14], auto-immune [15], and cardiovascular diseases [16], as well as cancers [17]. Moreover, cfDNA levels play a role as a predictor for mortality in middle-aged and old (46–76 years of age) individuals [18]. Although cfDNA is a non-specific marker, important requirements are met to test the hypothesis that cfDNA could be used as a marker for psychological stress. The cfDNA levels are affected by acute systemic inflammation in response to diseases [19] or physiological conditions, such as exercise [20], and show clear dose-response associations; cfDNA shows a lower within-subject variation compared to between-subject variation [21], and it can be measured highly precisely and reliably using cost-efficient qPCR approaches [22].

In the present study, it was examined whether the Trier Social Stress Test (TSST) acute social stress was sufficient to affect cfDNA levels in healthy young male participants using a controlled within-subject design. A homogenous cohort of healthy young subjects was chosen for the investigation of the kinetics of plasma cell-free DNA without any influence of psychopathology. These unaffected controls will serve as a control group, contrasting patient groups in further studies. Next to physiological parameters, such as cortisol and heart rate, self-report questionnaires were used to assess perceived stress and evaluate whether cfDNA kinetics reflects individual differences in stress response.

## 2. Materials and Methods

### 2.1. Study Participants

**Figure 1:**
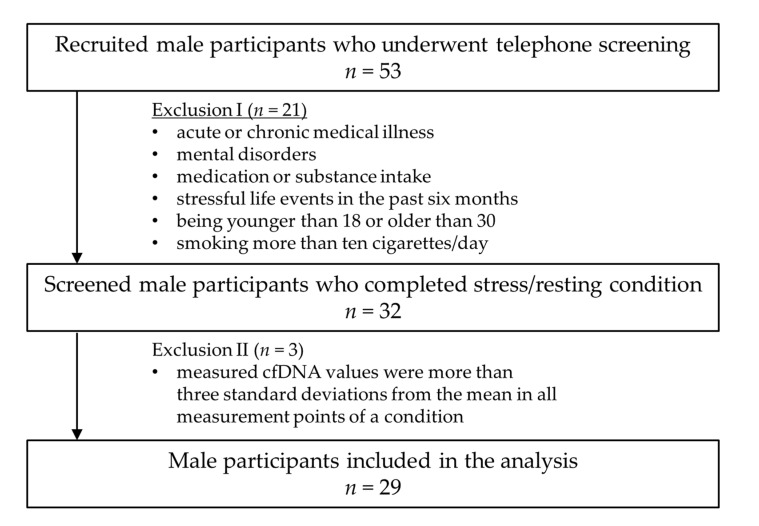
Sample inclusion/exclusion criteria flow chart.

The healthy male controls (*n* = 29) were recruited through electronic tendering (e-tendering) and notice boards at the Johannes Gutenberg University Mainz. The exclusion criteria (acute or chronic medical illness, mental disorders, medication or substance intake, stressful life events in the previous six months, being younger than 18 or older than 30 years of age, and smoking more than ten cigarettes/day) were checked in a telephone interview based on the entire procedure of the Structured Clinical Interview (SCID; [23]) for the Diagnostic and Statistical Manual of Mental Disorders (DSM-IV, [24]). A sample inclusion/exclusion criteria flow chart is given in Figure 1. The mean age of the twenty-nine male participants was 24.34 (SD = 4.08) with a mean BMI of 22.94 kg/m^2^ (SD = 1.61). Three of the 29 included participants were smokers (under 10 cigarettes per day). With regard to age- and gender-specific differences in cfDNA levels [25,26], only male participants of an age between 18 and 30 were included. All participants received an allowance of fifty euros after successful participation. The study protocol was approved by the local Ethics Committee of the Landesärztekammer Rheinland-Pfalz, Germany (No#2019-14188). A detailed description of all participants regarding demographic data and psychological status is given in Table 1.

### 2.2. Procedures

**Figure 2:**
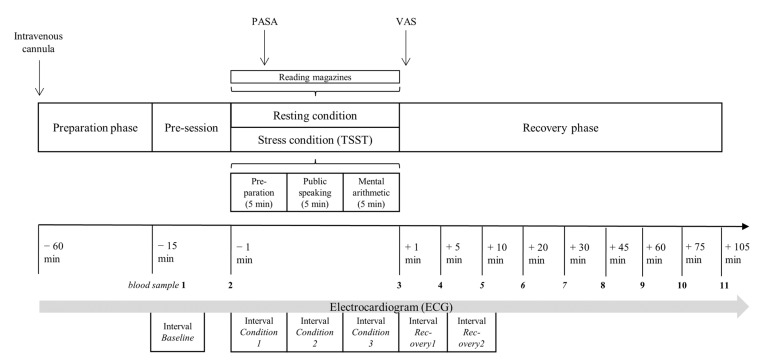
Overview of the examination procedure. PASA Primary Appraisal Secondary Appraisal, TSST Trier Social Stress Test, VAS Visual Analogue Scale.

The participants completed the stress and resting conditions on separate days over a time frame of seven days. The start of both conditions was scheduled between 2:00 p.m. and 5:00 p.m., and the testing sequence of the two conditions (stress and resting) was randomized. The participants were asked to refrain from eating, drinking, and smoking for at least two hours before testing and during the 2 h testing session. The intravenous cannula was inserted 45 min before the first blood sample was taken to avoid a pain-induced cfDNA release. The experimental protocol started with a 15-min pre-session, in which a three-minute respiratory sinus arrhythmia test (six breaths per minute) was performed, and two blood samples were collected. Afterwards, the healthy male adults went through the two 15-min conditions (stress and resting). The Trier Social Stress Test (TSST) was performed according to the published process protocol by Kirschbaum et al. [27], with three sections consisting of preparation, interview, and a calculation task (5 min per block). During the resting condition, the participants were given the opportunity to read magazines. The cognitive appraisal was evaluated three minutes after the start of either condition by the Primary Appraisal Secondary Appraisal (PASA; [28]). Immediately after both conditions, the self-reported stress perception of both conditions was measured with the visual analogue scale (VAS). After the stress and resting conditions, the participants were reposed in the supine position on a surgery bed for 105 min while nine blood samples (+1, +5, +10, +20, +30, +45, +60, +75, and +105 min) were collected. The participants were fitted with a chest trap to measure the heart rate during the whole experimental protocol with the Polar monitoring system V800 with the H10 sensor (Polar, Finland). An overview of the examination procedure is given in Figure 2.

### 2.3. Blood Analytics

For the determination of plasma cfDNA concentrations, blood samples were collected in monovettes containing ethylenediaminetetraacetic acid (EDTA) (Sarstedt; Nümbrecht; Germany). After blood sampling, the EDTA monovettes were directly immediately centrifuged at 4 °C and 2000× *g* for 10 min. The blood plasma was divided into aliquot tubes and stored at −80 °C. Quantification of plasma cfDNA is described in detail in Section 2.5. For the determination of serum cortisol concentrations, blood samples were collected in serum gel monovettes (S-Monovette^®^ 9 ml Z; Sarstedt; Nümbrecht; Germany). Monovettes were kept a room temperature for 30 min to enable blood coagulation. After coagulation, the serum monovettes were centrifuged for 10 min at 2500× *g* and 20 °C. Serum cortisol concentrations were determined using a commercially available enzyme-linked immunosorbent assay (ELISA) kit.

### 2.4. Psychological and Clinical Measures

The psychological and general status of the participants was measured with five instruments: (1) The Symptom-Check-List-90-R (SCL; [29]) is a self-report instrument for the evaluation of psychological and physical impairment, consisting of 90 items with a five-point rating scale (total score range 20–80). (2) The German version of the Perceived Stress Scale (PSS; [30]) contains 14 items using a five-point Likert scale that ranges from 1 (never) to 5 (very often). Depressive symptoms were recorded with (3) the Beck Depression Inventory (BDI; [31]), which includes 21 symptoms and attitudes that can be rated in terms of intensity from 0 to 3 (total score range: 0–63). (4) The participants also completed the Trier Inventory for Chronic Stress constructed by Schulz, Schlotz, and Becker [32] in order to assess their perceived chronic stress. (5) The Freiburg Questionnaire on Physical Activity (FFKA; [33]) was used to assess the different kinds of daily activities.

During and after the stress and resting conditions, two questionnaires were collected. The Primary Appraisal Secondary Appraisal (PASA; [28]) measured the cognitive appraisal processes. In the initial assessment, or primary appraisal, specific situations are evaluated as threatening or challenging. The second appraisal describes an individual’s perceived coping skills. A stress index is calculated as an integrated measure of transactional stress. A high stress index score indicates a higher general stress perception. The scale comprises 16 items to be rated on a six-point Likert scale ranging from 1 (completely disagree) to 6 (completely agree). The visual analogue scale (VAS) was used to assess the self-reported stress perception after both conditions and rates from 0 (no stress) to 100 (maximum stress).

### 2.5. Heart Rate Measurement

The heart rate was measured using the polar monitoring system V800 and the H10 sensor (Polar, Finland; sample rate: 1000 Hz). Based on the recorded R-R intervals, the heart rate was analyzed with Kubios software (Kubios Oy, Kuopio, Finland). An automatic filtering process method was applied to eliminate artifacts and extra beats in the R-R interval (threshold: 0.45 s). The heart rate was analyzed in the following 5 min intervals: condition 1 (0–5 min), condition 2, (5–10 min), condition 3 (10–15), recovery 1 (0–5 min after condition), and recovery 2 (5–10 min after condition).

### 2.6. Quantification of cfDNA

The cfDNA concentrations were determined as described by Neuberger et al. [22]. Ahead of the measurements, the linearity, limit of quantification, and limit of detection of the assay were determined. The qPCR assay amplifies DNA in unpurified plasma, which is diluted 1:10 in UltraPure DNase/RNase-Free H2O (Invitrogen, Waltham, MA), targeting a 90 bp fragment of human long interspersing nuclear elements (LINEs) of the repetitive L1PA2 family (5′-TGCCGCAATAAACATACGTG-3′ and 5′-GACCCAGCCATCCCATTAC-3′). Briefly, each sample was measured as a technical triplicate with 5 µL final volume containing 0.66 µL of 1:10 diluted plasma, 0.33 µL primer mix (140 nm final concentration of each primer) and 4 µL of qPCR mix with 0.6 U Velocity Polymerase (Bioline, London, UK), 1.2 × Hifi Buffer (Bioline, London, UK), 0.1 × SYBR Green (Sigma, St. Louis, MO, USA), and 0.3 mM dNTPs (Bioline, London, UK). The qPCR reaction was carried out using a CFX384 Bio-Rad (Bio-Rad, Munich, Germany) cycler with a two-step protocol. The cycling conditions were: initial heat activation at 98 °C for 2 min followed by 33 cycles of 95 °C for 10 s and 64 °C for 10 s with a subsequent melting curve from 70 to 95 °C with 0.5 °C increments for 10 s. The operator measuring the samples was blinded and did not know the allocation of the samples into the control or intervention group.

### 2.7. Statistical Analysis

All data analyses were performed using SPSS Statistics version 26 (IBM, Chicago, IL, USA). In view of a statistically meaningful sample size, the optimum statistical sample size was calculated with the G*power program (version: 3.1.9.2.) [34]. Based on the study by Hummel et al. [9], a large effect was found investigating the effect of a physical and psychosocial stressor on the cfDNA concentration. A power analysis showed that for a large effect size of Cohen’s f = 0.40, two testing conditions (TSST and resting condition), in each condition *n* = 11 repetitions, significance level of *p* = 0.05, and power of 80% (1−ß = 0.80), a total sample size of *n* = 30 participants for ANOVA-repeated measures (between factors) was needed. We tested 32 participants, but three participants were excluded from analyses regarding cfDNA concentration since their measured values were more than three standard deviations from the mean in all measurement points of a condition [35]. Single outliers of cfDNA concentration (more than or less than three standard deviations from the mean) were replaced with multiple imputation (number of single outliers *n* = 6 of *n* = 638 single cfDNA measurements). The data were analyzed according to the normality of distributions and were, in case of not normally distributed data, subjected to logarithm naturalis transformations.

First, the effects of the TSST and the resting condition over eleven measurements points for the cfDNA and cortisol concentration were analyzed with the ANOVA for repeated measurements to reveal possible main effect of condition or time and a possible time × condition interaction. The assumption of sphericity was controlled by Mauchly’s test. Whenever necessary, the ANOVA results were corrected with Greenhouse-Geisser. In addition, the area under the response curve (AUC) with respect to an increase in cfDNA and cortisol (AUC_I_) and the delta between peak (either +1, +5, +10, +20, +30, +45, +60, +75, +105 min sample) and baseline (Δ Peak-Base) were calculated (Fekedulegn et al., 2007). In order to compare the calculated values of both conditions the *t*-test for dependent means was used. For a specification of the association between cfDNA baseline and reactivity values in the stress condition, subgroups were formed by a median split of baseline cfDNA values from the TSST. The 29 healthy controls were categorized into low (cfDNA < 8.70 ng/mL, *n* = 14) and high (cfDNA > 8.70 ng/mL, *n* = 15) cfDNA baseline groups. The difference between the high and low cfDNA baseline groups in the derived stress reactivity parameters Δ Peak-Base with regard to the stress condition was tested by independent Student’s *t*-test.

Second, the success of the stress induction was evaluated by the heart rate reactivity. To reflect the effect of the stress condition on the heart rate, ANOVA for repeated measurements with within-factor condition (stress vs. resting) and time was analyzed. The delta between peak and baseline was calculated and the difference between the stress and the resting conditions was tested by the dependent *t*-test.

Third, to analyze the effect of stress induction on subjective stress appraisal, the cognitive appraisal processes (PASA) and the acute self-reported stress perception (VAS) of both conditions were compared by the *t*-test for dependent means (PASA and VAS).

Fourth, Pearson’s correlations were calculated to quantify the relationship between AUC_I_ (stress condition) and baseline concentration (stress/resting condition) of cfDNA, AUC_I_ (stress condition) and baseline concentration (stress/resting condition) of cortisol, and psychological measures (BDI, SCL-90, PSS, and TICS-SCSS).

## 3. Results

### 3.1. cfDNA Reactivity

**Figure 3:**
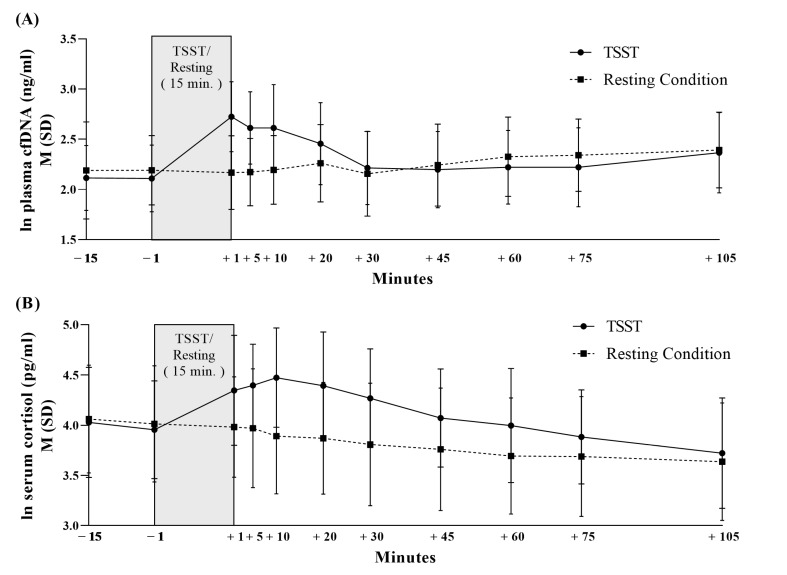
Cell-free DNA concentration (**A**) and cortisol concentration (**B**) during Trier Social Stress Test and resting condition in healthy men.

Figure 3 and Figure S1 present the cfDNA concentration over eleven measurement points during the resting and stress condition. There were no significant differences in the baseline cfDNA levels between the stress and resting condition (−15 min: *t* (28) = −0.993, *p* = 0.33; −1 min: *t* (28) = −1.498, *p* = 0.15). The 29 healthy male participants showed a 108% increase in the cfDNA concentration (Figure 1). ANOVA results indicated a significant effect of time over the eleven measurement points (*F* _(5.599, 156.769)_ = 19.061, *p* ≤ 0.001, *η2* = 0.405). Furthermore, a significant main effect of condition could be unveiled for cfDNA concentration with higher values for the stress induction condition compared to the resting condition (*F* _(1, 28)_ = 7.809, *p* ≤ 0.01, *η2* = 0.218) and a significant interaction effect time × condition (*F* _(5.818, 162.906)_ = 26.995, *p* ≤ 0.001, *η2* = 0.491). In regard to the derived parameters of the cfDNA concentration, there were significantly higher values of peak (*t* (28) = −2.914, *p* ≤ 0.01, *d* = −0.54), delta (*t* (28) = −4.192, *p* ≤ 0.001, *d* = −0.78), and AUC_I_ (*t* (28) = −3.712, *p* ≤ 0.001, *d* = −0.69) in the stress condition compared to the resting condition. In addition, baseline subgroups analysis revealed significantly higher cfDNA delta values from the stress condition in the high cfDNA baseline group than the low cfDNA baseline group (*t* (27) = −2.150, *p* ≤ 0.05, *d* = −0.80).

### 3.2. Cortisol Reactivity

As shown in Table 2 and Figure 3, there was a significant condition (*F* _(1, 27)_ = 34.918, *p* ≤ 0.001, *η2* = 0.564), time (*F* _(3.093, 83.512)_ = 46.272, *p* ≤ 0.001, *η2* = 0.632), and time × condition effect (*F* _(3.512, 94.883)_ = 17.324, *p* ≤ 0.001, *η2* = 0.391) in the cortisol concentration. In addition, significantly higher peak (*t* (27) = −7.046, *p* ≤ 0.001, *d* = −1.33), higher delta (*t* (27) = −6.471, *p* ≤ 0.001, *d* = −1.22), and higher AUC_I_ cortisol values (*t* (27) = −3.927, *p* ≤ 0.001, *d* = −0.74) in the stress condition compared to the resting condition could be unveiled. The unaffected controls showed no significant cortisol baseline differences between the resting and stress conditions (−15 min: *t* (27) = 0.297, *p* = 0.77; −1 min: *t* (27) = 0.387, *p* = 0.70).

### 3.3. Heart Rate Reactivity

**Figure 4:**
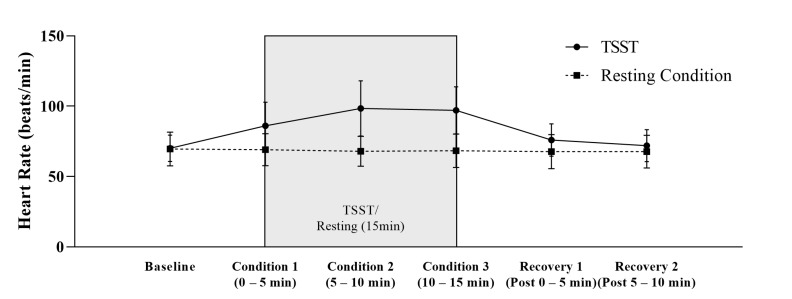
Heart Rate (M + SD) during Trier Social Stress Test and resting condition in healthy men (*n* = 23).

As shown in Table 2 and Figure 4, there was a significant condition (*F* _(1, 22)_ = 36.076, *p* ≤ 0.001, *η2* = 0.621), time (*F* _(1.827, 40.194)_ = 49.321, *p* ≤ 0.001, *η2* = 0.692), and time × condition effect (*F* _(1.951, 42.926)_ = 57.865, *p* ≤ 0.001, *η2* = 0.725) in the heart rate. In addition, significantly higher peak (*t* (22) = −7.584, *p* ≤ 0.001, *d* = −1.58) and delta (*t* (22) = −7.584, *p* ≤ 0.001, *d* = −1.58) heart rate values in the stress condition compared to the resting condition could be unveiled. The unaffected controls showed no significant heart rate baseline difference between the resting and stress conditions (*t* (22) = −0.260, *p* = 0.80).

### 3.4. Stress Appraisal

All participants exhibited a significant higher stress perception in the VAS after the TSST compared to the resting condition (*t* (28) = −8.903, *p* ≤ 0.001, *d* = −1.65). The tertiary scale ‘stress index’ of the anticipatory cognitive appraisal of stress (PASA) demonstrated significantly lower values before the TSST than during the resting condition (*t* (28) = −7.663, *p* ≤ 0.001, *d* = −1.42). In addition, all primary scales with the exception of the scale ‘self-concept’ and both secondary scales of the PASA showed significant difference between stress and resting conditions (see Table 2).

### 3.5. Associations between Psychological and cfDNA and Cortisol Measures

No significant correlation could be found between psychological (BDI, SCL-90, PSS, and TICS-SCSS), cfDNA, and cortisol measures (see Table 3).

## 4. Discussion

Acute psychological stress affects the immune system and activates peripheral inflammatory pathways [8]. Here, we explored the responsiveness of cfDNA to acute stress using TSST in a controlled within-subject design. The TSST is a standardized and well-established test and induces a strong psychological stress response affecting autonomic, endocrinological and immunological activity [27]. The present findings show that a brief psychological stressor was sufficient to evoke robust and rapid increases in the cfDNA. No cfDNA differences were detected in the controlled resting condition. Our study with healthy young subjects did not reveal an association between the cfDNA and perceived stress as measured by self-reported questionnaires. Interestingly, subjects with higher basal levels of cfDNA showed stronger cfDNA increases in response to the TSST compared to subjects with lower levels. The current findings identify cfDNA as a factor that contributes to the psychobiological effect of stress and provide further evidence for stress-related increases in cfDNA.

A growing body of evidence underlines that pathophysiological conditions, such as cancer, are accompanied by elevated cfDNA levels [17], older individuals show increased values [36], and cfDNA levels are associated with overall mortality [18]. Until now, only two studies had evaluated the kinetics of cfDNA in response to acute stress showing conflicting results [9,37]. Hummel et al. [9], studied the concentration of plasma cfDNA and mitochondrial DNA (mtDNA) in response to the TSST and found ~2 fold-increases in the cfDNA. The results are consistent with our findings showing that acute stress increases cfDNA concentration 108% and decreasing to baseline levels 30 min after TSST. In the control setting, the cfDNA levels remained at pre-concentration levels. In contrast to this, Trumpff et al. [37], who studied the effect of acute stress on mtDNA and cfDNA, did not find increased nuclear cfDNA in the serum samples of healthy middle-aged participants, but only increases in mtDNA [37]. However, notably, plasma cfDNA levels better represent the in vivo levels of cfDNA since serum samples typically show higher basal cfDNA levels, which are related to the clotting process [38]. Notably, the centrifugation speed of plasma samples can have a large effect on the concentration of mtDNA [39], whereas the cfDNA concentration does not differ relevantly between platelet-free compared to platelet-rich plasma samples [22].

With regard to the well-established stress biomarkers cortisol [40] and heart rate [41], we did not find any significant associations between cfDNA, cortisol, and heart rate baseline values and stress reactivity. In line with this, the study by Hummel et al. [9] also demonstrated no association between cortisol and cfDNA stress reactivity in response to an acute psychosocial stressor. Regarding heart rate, high stress responders of ccf-mtDNA revealed higher baseline heart rate, but no differences in heart rate reactivity [37]. Cortisol is part of the neuroendocrine-immune activation [42], heart rate displayed the role of sympathetic regulation of immune responses [43], and cfDNA has a physiological regulator role related to the innate immunity [44]. There is cross-talk between these different systems [42], but no evidence that cortisol, heart rate, and cfDNA are primary mediators in the link between these systems.

Moreover, our results did not indicate associations with perceived stress levels as measured by the Perceived Stress Scale and the Trier Inventory for Chronic Stress, which might be related to the small homogenous cohort of healthy young subjects. This suggests that alterations in cfDNA and cortisol might reflect a more long-lasting psychopathology. There is evidence of a blunted cortisol reactivity in individuals with panic disorders or depression compared to healthy controls [45,46]. Qi et al. [10] studied cfDNA serum levels in patients with schizophrenia, major depressive disorder (MDD), and alcohol-induced psychotic disorder (AIPD) compared to healthy subjects. Schizophrenia patients showed significantly higher basal cfDNA levels compared to healthy persons. The values were ~ 2-fold higher increased. The cfDNA levels of MDD and AIPD patients did not differ from healthy cohort, whereas receiver operating characteristic curves analysis indicated effectiveness for complementary diagnosis between schizophrenia and MDD and AIPD patients [10]. Further research is required to evaluate if chronic stress levels are associated with increased basal levels of cfDNA, which might be indicative for stress-related disorders. Dichotomizing our study cohort in a subject with high or low basal levels (median split) revealed that persons with a higher cfDNA concentration showed significantly higher increases of cfDNA in response to acute stress.

Notably, our qPCR quantification assay targets a LINE-1 element. These long-interspersing elements type 1 (LINE-1) are retrotransposon, which comprises ~17% of the human genome. Most of the LINE-1 elements are inactive and cannot move to new positions [47]. However, it is estimated that ~100 copies are still retro-transposition-competent [48,49]. Recent evidence suggests that LINE-1 activity contributes to age-related diseases, such as neurodegeneration and cancer. Moreover, LINE-1 methylation level, sequence variation, and activity have been reported in other disorders, including depression, schizophrenia, and bipolar disorders [50,51,52]. According to the high number of copies per genome of our assay (3416), and the relative rare event of retrotransposition, a relevant physiological bias is unlikely. However, the relative amount of LINE-1 copies compared to single gene copies could be analyzed in cohort studies comparing healthy and diseased persons.

The origin of cfDNA, factors triggering its release into the circulation as well as the functional properties of cfDNA are subject to intense investigation. Moss et al. [12] analyzed the genome-wide methylation pattern of cfDNA. The authors identified hematopoietic cells to be the major source cells, whereas granulocytes showed the highest percentage with ~32% of the DNA [12]. The origin of cfDNA during acute stress has been unknown until recently. Hummel et al. [9] studied the methylation status of a single locus of homeobox A5 gene (HOXA5). The authors found different methylation profiles after acute psychological stress compared to physiological stress [9]. This might indicate different release mechanisms, whereby further research is required to identify the source cells of cfDNA in response to acute or chronic stress. During exercise as a physiological stress situation, cfDNA is almost exclusively released from granulocytes [53], whereas the activation of platelets, catecholamine release, and mechanical and thermal stress are discussed as being putative triggers [54]. Although small extracellular vesicles, or exosomes, were considered to relevantly contribute to DNA release, only a minor fraction of cfDNA is associated with EVs, indicating that EVs are of minor importance for circulatory cfDNA increases [55,56].

In an exploratory pilot study, Czamanski-Cohen et al. [11] provided the first evidence that cfDNA levels might be responsive to stress reduction techniques. The researchers studied 14 women undergoing in vitro fertilization. Women who engaged in stress reduction techniques showed reduced plasma cfDNA levels [11]. In critical pathological situations, such as sepsis, a clear proportional association can be detected between the cfDNA release and the severity of the systemic inflammation [57]. Our data provide support for a possible association between stress-related persistent increasing cfDNA levels and subclinical inflammation. However, further research is required to study if interventional programs might show responses on the cfDNA level. Moreover, in vitro experiments indicated that cfDNA is an essential immune system regulator triggering the release of pro-inflammatory cytokines [44], whereby more studies are warranted to clarify the functional physiological role of cfDNA. 

Several limitations in the current study should be pointed out. Despite the fact that cfDNA levels are affected by acute systemic inflammation, the main limitation of this study is the non-assessment of well-defined inflammatory markers, such as cytokines IL-6, IL-10, IL-ß, and TNF-a. With regard to the quantification of plasma cfDNA, a single centrifugation step at 2000 g is not sufficient to deplete platelets that contain a relevant amount of mitochondrial DNA (mtDNA). With respect to the analysis of mtDNA, the centrifugation protocol would provoke a huge bias [39]. However, we (and others) studied the effect of different centrifugation protocols (1 × 600× *g* vs. 2 × 2500× *g*) and were able to show that the concentration of nuclear cfDNA is not affected [22,39]. Due to age- and gender-specific differences in cfDNA levels [25,26], only male participants of an age between 18 and 30 were included in the study. Therefore, it must be considered that the significance of the findings is restricted only to unaffected male controls and a transfer to women might be of interest as well.

## 5. Conclusions

In conclusion, our findings show that cfDNA is a dynamic stress-inducible marker that is affected by acute psychological stress. Further research is required to evaluate if cfDNA might be a valid marker for displaying the psychological stress response and neuro-endocrine-immune activation, maybe even identifying individuals who are vulnerable to mental disorders, or if cfDNA diagnostics could be used for monitoring treatment progression.

## Figures and Tables

**Table 1:** Characteristics of the male participants.

	Individuals (*n* = 29)
Demographic data	
Age (years), M (SD), [Min–Max]	24.34 (4.08), [19–34]
Body mass index, M (SD), [Min–Max]	22.94 (1.61), [19.94–26.32]
Smoking, *n* (%)	3 (10.4)
FFKA—Total activity (min/week), M (SD), [Min–Max]	1534 (1047), [246–4230]
FFKA—Sport activity (min/week), M (SD), [Min–Max]	473 (583), [0–2700]
Psychological Assessment	
BDI, M (SD), [Min–Max]	5.62 (4.67), [0–15]
SCL Global Severity Index, M (SD), [Min–Max]	0.36 (0.29), [0.01–1.17]
PSS, M (SD), [Min–Max]	22.24 (6.78), [7–36]
TICS-SCSS, M (SD), [Min–Max]	12.10 (6.26), [3–28]

BDI Beck Depression Inventory, FFKA Freiburg Questionnaire on Physical Activity, M mean, PSS Perceived Stress Scale, SCL Symptom-Check-List-90-R, SCSS Subscale of Chronic Stress, SD Standard Deviation, TICS Trier Inventory of Chronic Stress.

**Table 2:** Derived cfDNA parameters, heart rate parameters and subjective appraisal with respect to conditions.

Healthy Men (*n* = 29)							
		Resting Condition		TSST		Dependent *t*-Test	
Derived cfDNA Parameters		M	SD	M	SD	*t*	*p*
Peak		14.25	4.58	17.92	7.08	−2.914	≤0.01 ** (*d* = −0.54)
Delta _Peak-Baseline_		4.75	2.92	9.23	5.10	−4.192	≤0.001 *** (*d* = −0.78)
AUC_I_		102.17	260.75	306.70	198.08	−3.712	≤0.001 ** (*d* = −0.69)
**Derived cortisol parameters**							
Peak ^a)^		63.09	17.90	94.34	23.26	−7.046	≤0.001 *** (*d* = −1.33)
Delta _Peak-Baseline_^a)^		10.74	8.69	42.35	25.87	−6.471	≤0.001 *** (*d* = −1.22)
AUC_I_^a)^		−1168.22	1299.72	930.13	2457.85	−3.927	≤0.001 *** (*d* = −0.74)
**Derived heart rate parameters**							
Peak ^b)^		70.44	11.64	100.59	18.85	−7.584	≤0.001 *** (*d* = −1.58)
Delta _Peak-Baseline_^b)^		0.88	3.20	31.04	20.20	−7.584	≤0.001 *** (*d* = −1.58)
**Subjective Appraisal**							
PASA	Threat, (1–6)	1.47	0.83	3.09	1.08	−7.855	≤0.001 *** (*d* = −1.46)
	Challenge, (1–6)	2.35	0.96	4.07	0.79	−8.100	≤0.001 *** (*d* = −1.50)
	Self concept, (1–6)	4.55	0.89	3.91	1.02	2.660	≤0.05 * (*d* = 0.49)
	Control expectancy, (1–6)	4.63	0.73	4.31	0.84	2.002	0.055 (*d* = 0.49)
	Primary appraisal, (1–6)	1.91	0.79	3.58	0.82	−9.529	≤0.001 *** (*d* = −1.77)
	Secondary appraisal, (1–6)	4.59	0.64	4.11	0.79	3.143	≤0.001 *** (*d* = 0.58)
	PASA—Stress index	−2.68	1.28	−0.54	1.39	−7.663	≤0.001 *** (*d* = −1.41)
VAS		41.35	11.23	58.51	9.42	−8.903	≤0.001 *** (*d* = −1.65)

AUC_I_ area under the curve with respect to increase, M Mean, PASA Primary Appraisal Secondary Appraisal, SD Standard Deviation, VAS Visual Analogue Scale. ^a)^ Sub-sample of *n* = 29 participants ^b)^ Sub-sample of *n* = 23 participants. *: *p* ≤ 0.05, **: *p* ≤ 0.01, ***: *p* ≤ 0.001.

**Table 3:** Pearson’s correlations (r) between psychological, cfDNA, and cortisol measures.

		ln Baseline cfDNA		cfDNA AUC_I_	ln Baseline Cortisol ^a)^		Cortisol AUC_I_^a)^	Psychological Measures			
		Resting Condition	Stress Condition	Stress Condition	Resting Condition	Stress Condition	Stress Condition	BDI	SCL-GSI	PSS	TICS-SCSS
ln baseline cfDNA	Resting condition	1.00									
	Stress condition	0.63, *p* ≤ 0.001	1.00								
cfDNA AUCI	Stress condition	0.23, *p* = 0.23	0.41, *p* ≤ 0.05	1.00							
ln baseline cortisol ^a)^	Resting condition	0.13, *p* = 0.52	0.09, *p* = 0.66	−0.04, *p* = 0.83	1.00						
	Stress condition	0.07, *p* = 0.74	0.15, *p* = 0.44	0.11, *p* = 0.58	0.28, *p* = 0.15	1.00					
cortisol AUCI ^a)^	Stress condition	−0.01, *p* = 0.95	0.06, *p* = 0.75	0.18, *p* = 0.35	0.11, *p* = 0.57	0.59, *p* ≤ 0.01	1.00				
Psycho-logical measures	BDI	−0.01, *p* = 0.95	−0.21, *p* = 0.28	−0.16, *p* = 0.41	0.24, *p* = 0.23	0.00, *p* = 0.99	−0.26, *p* = 0.18	1.00			
	SCL-GSI	0.01, *p* = 0.95	−0.15, *p* = 0.43	−0.10, *p* = 0.60	0.19, *p* = 0.33	0.06, *p* = 0.76	−0.22, *p* = 0.26	0.74, *p* ≤ 0.001	1.00		
	PSS	0.07, *p* = 0.73	0.27, *p* = 0.15	0.04, *p* = 0.85	−0.04, *p* = 0.85	0.19, *p* = 0.33	−0.34, *p* = 0.08	0.47, *p* ≤ 0.01	0.46, ≤ 0.05	1.00	
	TICS-SCSS	−0.18, *p* = 0.35	−0.01, *p* = 0.97	0.16, *p* = 0.40	−0.23, *p* = 0.25	0.12, *p* = 0.54	−0.21, *p* = 0.28	0.27, *p* = 0.17	0.49, *p* ≤ 0.01	0.65, *p* ≤ 0.001	1.00

Data are presented as coefficient, *p* values. AUC_1_ area under the curve with respect to increase, BDI Beck Depression Inventory, GSI—Global Severity Index, PSS Perceived Stress Scale, SCL Symptom-Check-List-90-R, SCSS Subscale of Chronic Stress, TICS Trier Inventory of Chronic Stress, ^a)^ Sub-sample of *n* = 29 participants.

## Data Availability

Not applicable.

## Supplementary Materials

The following supporting information can be downloaded at: https://www.mdpi.com/article/10.3390/cells12040564/s1, Figure S1: Spaghetti plot of cell free DNA concentration (A,B) and cortisolconcentration (C,D) during Trier Social Stress Test and resting condition.
