# Supplementary material for: Kinetics of Plasma Cell-Free DNA under a Highly Standardized and Controlled Stress Induction
Source: Cells. 2023 Feb 9;12(4):564. doi: 10.3390/cells12040564 (PMC9954572; doi:10.3390/cells12040564)
Supplement: Supplementary file 1 [file cells-12-00564-s001.zip › cells-2118691-supplementary.pdf]

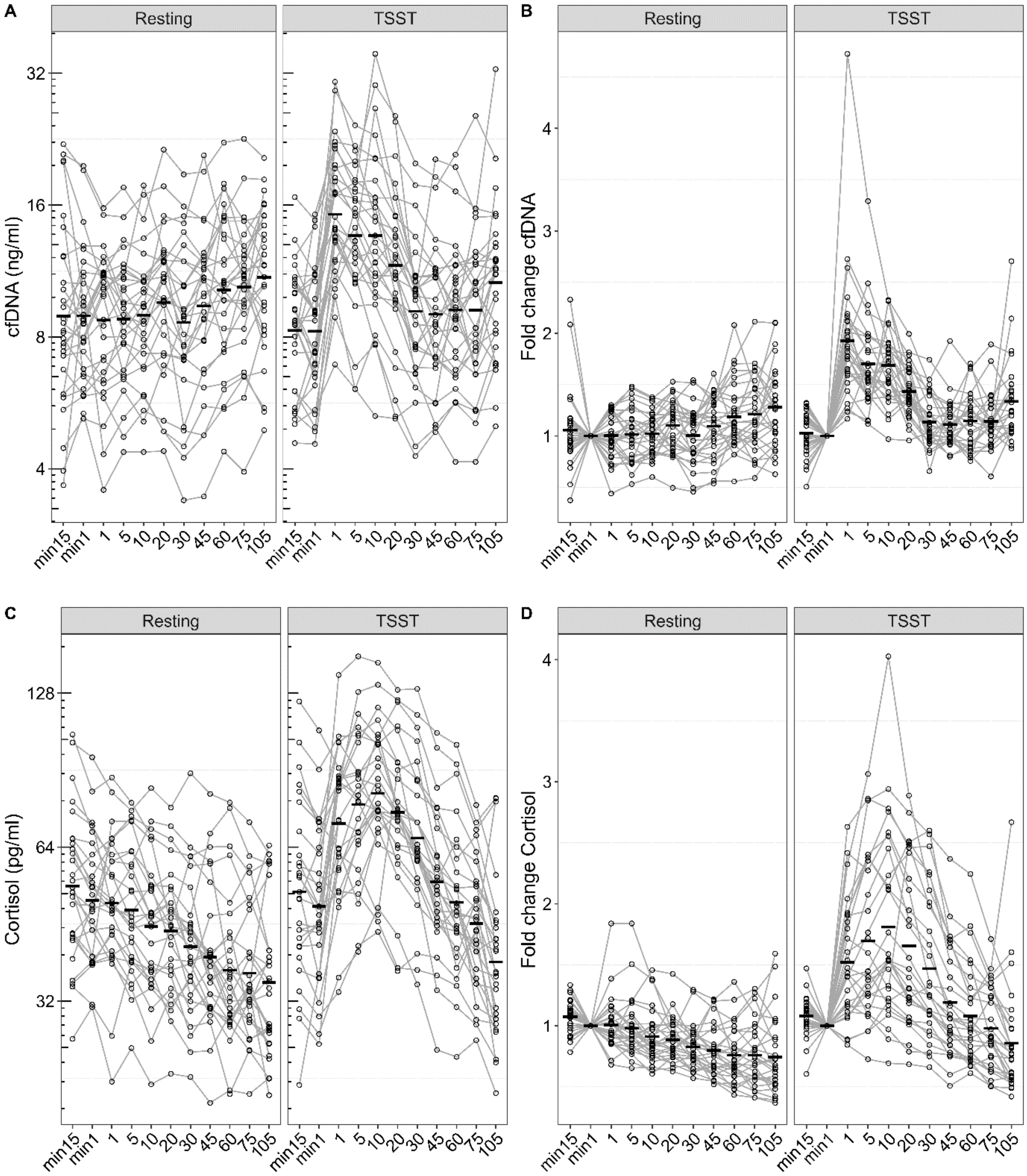

Supplemental Figure S1. Spaghetti plot of cell-free DNA concentration (A,B) and cortisol concentration (C,D) during Trier Social Stress Test and resting condition.
